# Characterization of aberrant pathways activation and immune microenviroment of BK virus associated nephropathy

**DOI:** 10.18632/aging.103486

**Published:** 2020-07-13

**Authors:** Yongguang Liu, Song Zhou, Jianmin Hu, Wentao Xu, Ding Liu, Jun Liao, Guorong Liao, Zefeng Guo, Yuzhu Li, Siqiang Yang, Shichao Li, Hua Chen, Ying Guo, Ming Li, Lipei Fan, Liuyang Li, Anqi Lin, Ming Zhao

**Affiliations:** 1 Department of Organ Transplantation, Zhujiang Hospital, Southern Medical University, Guangzhou, Guangdong, China

**Keywords:** BK virus nephropathy, immune microenvironment, renal transplant

## Abstract

In the context of transplantation with the use of immunosuppressive drugs, BK virus infection has become the main cause of BK virus nephropathy(BKVN) in renal transplant recipients(KTRs). More importantly, BKVN may cause further allograft dysfunction and loss. However, the role of the immune microenvironment in the pathogenesis of BKVN remains unknown. Therefore, we collected microarray data of KTRs to elucidate the immune characteristics of BKVN. Via the CIBERSORT, we found that BKVN had relatively more activated memory CD4 T cells. Immunostaining showed that CD4+ and CD8+cells were significantly different between BKVN and stable allografts(STAs). In addition, the expression of immune-related genes(antigen presentation, cytotoxicity, and inflammation) was significantly higher in BKVN than in STAs. The results of gene set enrichment analysis(GSEA) and single-sample GSEA(ssGSEA) indicated that immune cell-,cytokine-,chemokine-, and inflammation-related pathways were significantly activated in BKVN, while metabolism- and renal development-related pathways were significantly downregulated in BKVN. In addition, the immune microenvironments of the peripheral blood in patients with BK viremia(BKV) or transplant kidney biopsy(TKB) with BKVN may be different. Overall, the immune microenvironment may play important roles in the occurrence and development of BKVN and provide a theoretical basis for preventing the occurrence of BKVN and finding novel treatments.

## INTRODUCTION

BK virus is a nonenveloped, double-stranded DNA (dsDNA) Polyomaviridae virus that usually infects children. BK virus remains dormant and does not cause significant issues in healthy individuals; > 80% of adults are seropositive for BK viremia (BKV) [1]. BK virus infection has become the main cause of BK virus nephropathy (BKVN) in renal transplant recipients (KTRs) after renal transplantation with the use of immunosuppressive drugs [1, 2]. Cell lysis, necrosis and renal interstitial fibrosis are the main features of BKVN, and the incidence of allograft loss and failure caused by BKVN is approximately 10%-80% [3–5]. At present, the widespread use of immunosuppressants (such as calcineurin inhibitors (tacrolimus) and antiproliferative agents (mycophenolate acid) is considered to be the most important risk factor for BKV activation and replication [6].

To date, there are no effective antiviral drugs against BK virus infection, and reducing the doses of immunosuppressive agents has become the consensus approach, but the relationship between dose reduction and an increased risk of acute rejection must be carefully weighed [1, 7, 8]. Currently, few randomized, controlled trials are available to guide the treatment of BKV and BKVN in KTRs [2, 9]. Retrospective studies have shown that leflunomide and intravenous immunoglobulin may decrease the BK viral load and have potential clinical benefits, but further prospective, controlled studies are needed to confirm the efficacy and safety of these drugs for BKV and BKVN treatment. Therefore, exploring the pathogenesis of BKVN to identify more treatment approaches is particularly important.

Recent studies have shown that innate and adaptive immunity may play important roles in the pathogenesis of BKVN. Hammer et al. showed that an increased BK viral load was associated with a significantly upregulated BKV-specific CD8+ T cell level [10]. In addition, KTRs with BKV-specific CD8+ T cells eventually lost their grafts [10]. The interactions of NKG2DR on NK cells (NKs) with pentraxin 3 (PTX3) and MICB were also related to BKVN [11]. Proinflammatory cytokines are also involved in the pathogenesis of BKVN; for example, high expression levels of interleukin (IL)-6, IL-8, CCL5, CCL9, CCL10 and MCP-1 are associated with BKVN [7]. In addition, fibrosis of the transplanted kidney in BKVN patients is related to increased expression of TGF-β, MMP2 and MMP9 [1, 12]. However, no research has been systematically conducted to analyze the differences in immune cell infiltration, pathways, and immune-related gene expression between BKVN and stable allografts (STAs).

Overall, we tried to assess the infiltrating immune cells, immune-related features, and pathway activation levels in BKVN, STA, and BKV and to clarify the immune feature differences and connections among BKVN, STA, and BKV. Additionally, we attempt to depict the potential immunological mechanisms underlying the occurrence and development of BKVN, which may help to provide new strategies for the management of BKVN.

## RESULTS

### Differences in immune cells in the renal parenchyma between BKVN and STAs

**Figure 1:**
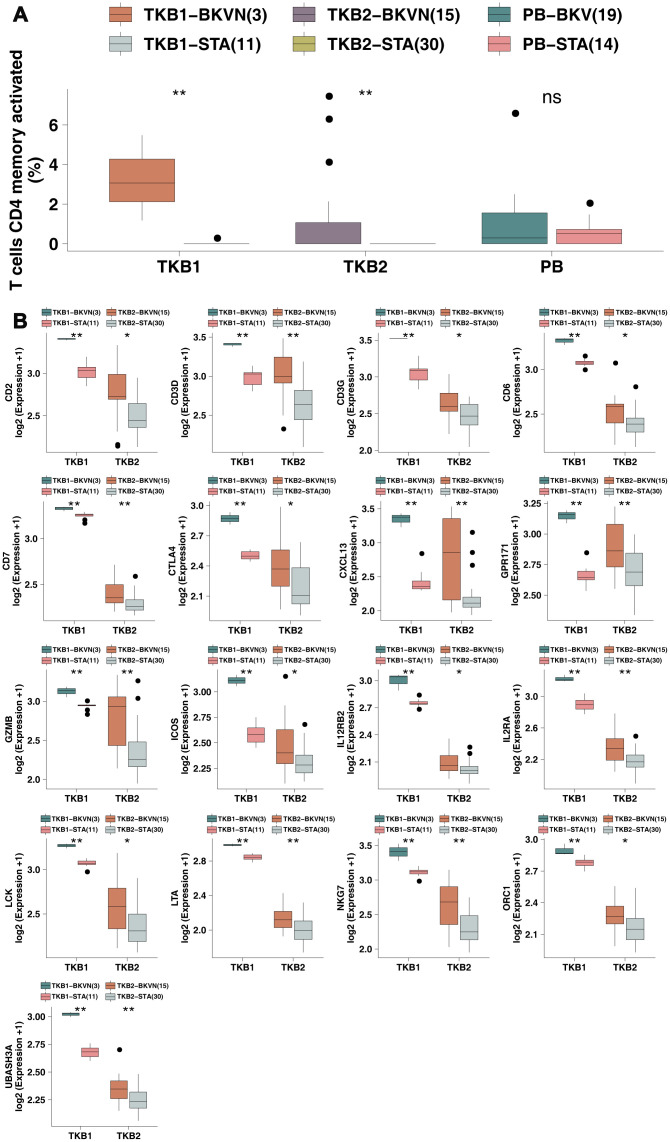
**BKVN was associated with increased levels of activated memory CD4 T cells and their cell markers.** (**A**) The proportions of activated memory CD4 T cells in the PB (STA and BKV), TKB1 (STA and BKVN) and TKB2 (STA and BKVN) datasets. (**B**) Comparison of the expression of cell markers of activated memory CD4 T cells between TKB1 (STA and BKVN) and TKB2 (STA and BKVN). The thick line represents the median value. The bottom and top of the boxes indicate the 25^th^ and 75^th^ percentiles (interquartile range). The whiskers encompass 1.5 times the interquartile range. *, P< 0.05; **, P< 0.01; ***, P< 0.001; and ****, P<0.0001; Mann-Whitney U test.

**Figure 2:**
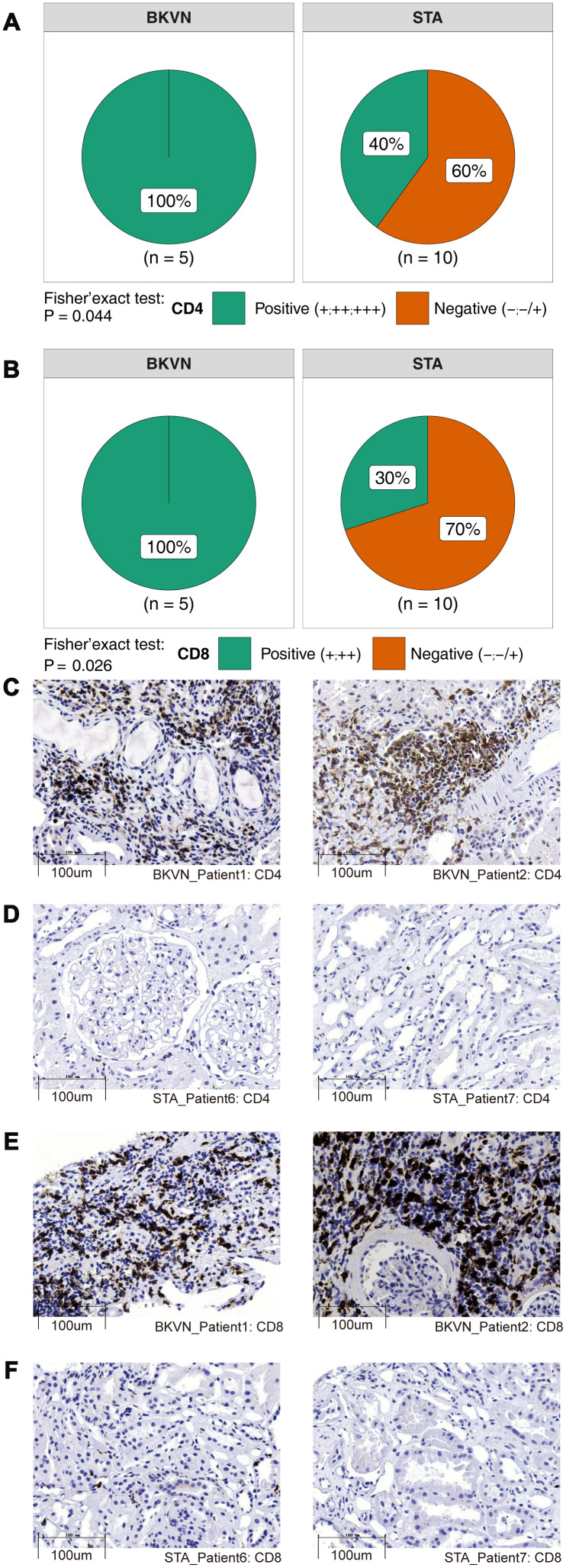
**Comparison of immunohistochemical characterization of the CD4+ and CD8+ cells between BKVN and STA.** (**A**, **B**) BKVN was associated with increased CD4+ (**A**) and CD8+ cells (**B**). (Fisher's exact test). (**C**–**F**) Immunohistochemical features (CD4 and CD8) of BKVN and STA.

Via the CIBERSORT algorithm, we analyzed the fractions of immune cells in TKB1, TKB2, and PB based on different BK virus infection statuses (Supplementary Figure 2). Figure 1A shows that compared with TKB1-STA renal parenchyma, TKB1-BKVN renal parenchyma has higher infiltration of activated memory CD4 T cells [3.10% (2.10%-4.30%) versus 0 (0-0), respectively; P <0.01]. Similarly, activated memory CD4 T cells exhibited significantly more infiltration into the renal parenchyma of TKB2-BKVN than TKB2-STA [mean: 1.3% versus 0, respectively; P <0.01], whereas no statistically significant differences were observed with regard to the PB samples of KTRs (PB-BKV versus PB-STA). We used immunohistochemistry to detect CD4+ and CD8+ cells. Noticeably, the differences in the level of CD4+ cells among the two groups were statistically significant (P<0.05; Figure 2A). Additionally, the CD8 markers in the BKVN of all the patients were positive (P<0.05; Figure 2B). Furthermore, CD4 and CD8 were regularly detected in the BKVN rather than STA (Figure 2C–2F).

To this end, we continued to analyze differences in the expression of cell markers of activated memory CD4 T cells (reported by Newman et al. [13]) in the renal parenchyma between TKB-STA and TKB-BKVN (Figure 1B). We found that both TKB1-BKVN and TKB2-BKVN had higher expression of activated memory CD4 T cell marker genes (all P <0.05), including Cluster of Differentiation (CD) genes, such as CD2, CD3D, CD3G, CD6, and CD7; CXCL13 (chemokine related); GPR171; GZMB (CYT related); ICOS; IL12RB2; ILRA; LCK; LTA; NKG7; ORC1; and UBASHA3.

### Differences in immune cells in the PB between BKV and STAs

**Figure 3:**
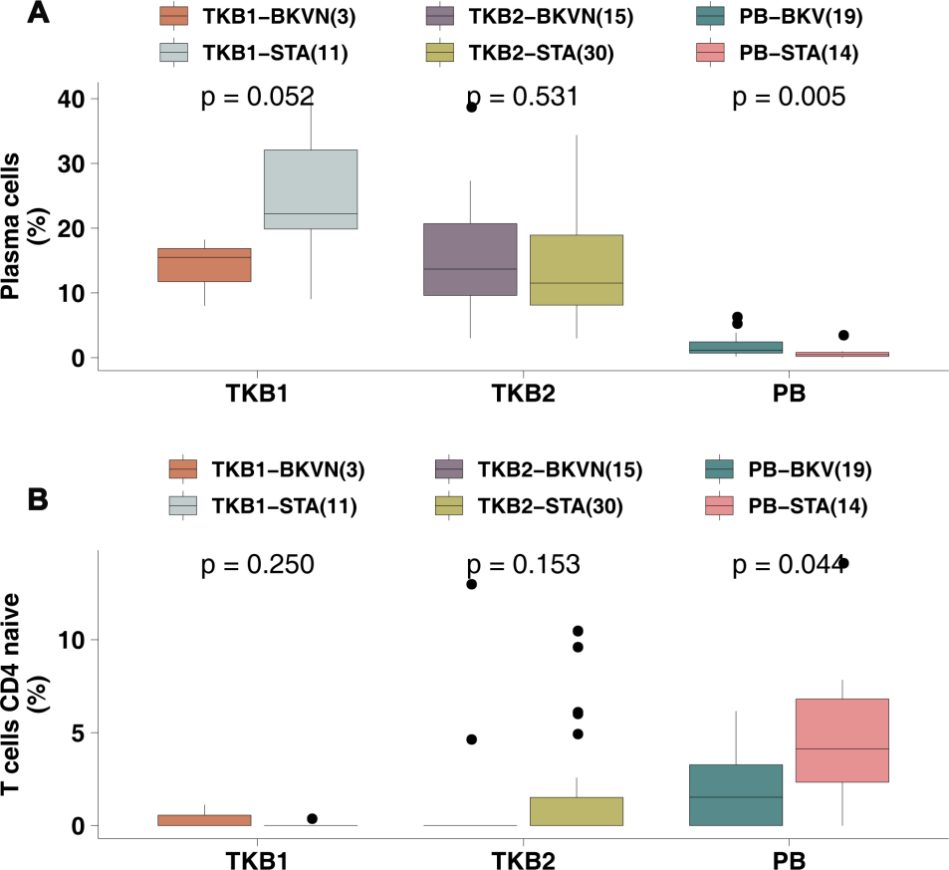
**BKV was associated with increased plasma cell numbers and decreased naive CD4 T cell numbers.** (**A**) The proportions of plasma cells in the PB (STA and BKV), TKB1 (STA and BKVN) and TKB2 (STA and BKVN) datasets. (**B**) The proportions of naive CD4 T cells in the PB (STA and BKV), TKB1 (STA and BKVN) and TKB2 (STA and BKVN) datasets. The thick line represents the median value. The bottom and top of the boxes indicate the 25^th^ and 75^th^ percentiles (interquartile range). The whiskers encompass 1.5 times the interquartile range. *, P< 0.05; **, P< 0.01; ***, P< 0.001; and ****, P<0.0001; Mann-Whitney U test.

To explore the differences in the proportions of immune cells in the PB between BKV and STAs, we used the Wilcoxon rank-sum test to compare the fractions of immune cells between PB-BKV and PB-STA and found that PB-BKV had higher infiltration of plasma cells [12.00% (7.00%-24.00%) versus 0 (0-0), respectively; P <0.01; Figure 3A] and fewer naive CD4 T cells [1.50% (0-3.30%) versus 4.1% (2.3%-6.8%), respectively; P <0.05; Figure 3B]. In contrast, in the TKB1 and TKB2 groups, the proportions of these cells were not significantly different between BKVN and STAs.

To this end, we continued to analyze the differences in the expression of plasma cell and naive CD4 T cell markers (reported by Newman et al. [13]) in PB samples between PB-STA and PB-BKV. For the cell markers of plasma cells, PB-BKV gene expression levels of IGKC and MZB1 were significantly higher than those of PB-STA (Supplementary Figure 3A; all P <0.05). Similarly, for naive CD4 T cell markers, the gene expression levels of CD2, CD247 and ZAP70 were significantly higher in the PB-BKV group than in the PB-STA group (Supplementary Figure 3B; all P <0.05).

### Differences in the expression of immune-related genes

**Figure 4:**
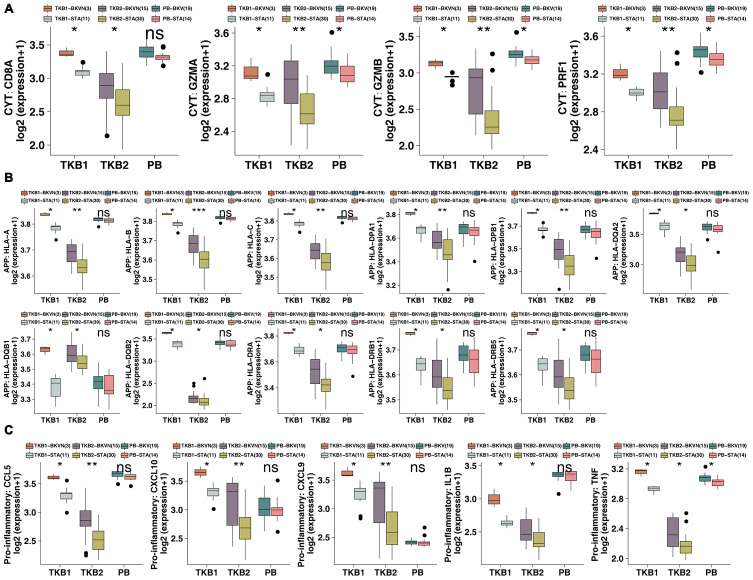
**BKVN was associated with increased expression of immune-related genes.** (**A**) The expression of CYT-related genes in the PB (STA and BKV), TKB1 (STA and BKVN) and TKB2 (STA and BKVN) datasets. (**B**) The expression of APP-related genes in the PB (STA and BKV), TKB1 (STA and BKVN) and TKB2 (STA and BKVN) datasets. (**C**) The expression of proinflammatory-related genes in the PB (STA and BKV), TKB1 (STA and BKVN) and TKB2 (STA and BKVN) datasets. The thick line represents the median value. The bottom and top of the boxes indicate the 25^th^ and 75^th^ percentiles (interquartile range). The whiskers encompass 1.5 times the interquartile range. *, P< 0.05; **, P< 0.01; ***, P< 0.001; and ****, P<0.0001; Mann-Whitney U test.

Next, we collected immune-related gene sets (including APP-, CYT-, chemokine-, and cytokine-related genes [14, 15]) to compare differential expression between TKB-BKVN and TKB-STA and between PB-BKV and PB-STA. The expression of CYT-related genes, such as CD8A, GZMA, GZMB and PRF1, in TKB1-BKVN was significantly higher than that in TKB1-STA (all P <0.05; Figure 4A). Similarly, TKB2-BKVN had significantly higher expression of CYT-related genes than TKB2-STA (all P <0.05; Figure 4A). Similarly, the expression of CYT-related genes in PB-BKV was significantly higher than that in PB-STA (all P <0.05; Figure 4A). The expression of APP-related genes in TKB-BKVN (either the TKB1 or TKB2 group) was significantly higher than that in TKB-STA (all P <0.05; Figure 4B), whereas there were no significant differences in the expression of APP-related genes between PB-BKV and PB-STA (Figure 4B). Figure 4C shows that the expression of chemokine- and cytokine-related genes, such as CCL5, CXCL9, CXCL10, IL1B and tumor necrosis factor (TNF), in TKB-BKVN was significantly higher than that in TKB-STA, whereas there were no significant differences in the expression of these genes between PB-BKV and PB-STA (Figure 4C).

### Differences in enriched pathways in the renal parenchyma between BKVN and STA

**Figure 5:**
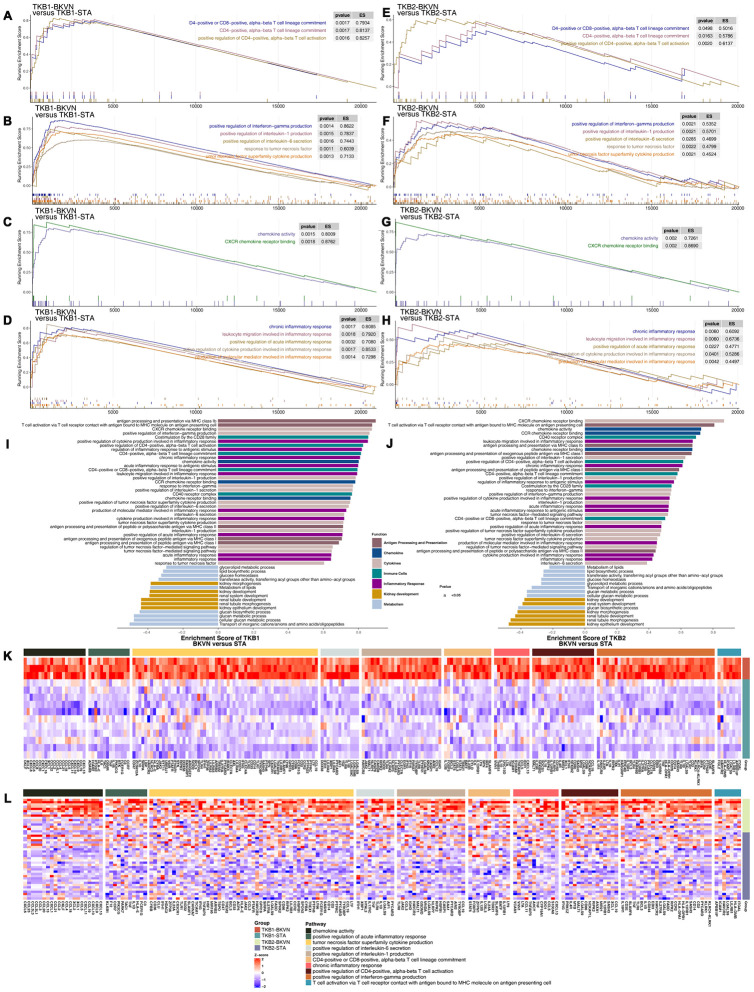
**GSEA of hallmark gene sets in TKB-BKVN and TKB-STA data downloaded from MSigDB (GSE47199 and GSE75693).** TKB1-BKVN was associated with activated immune cell- (**A**), cytokine- (**B**), chemokine- (**C**) and inflammation-related pathways (**D**). Similarly, TKB2-BKVN was associated with activated immune cell- (**E**), cytokine- (**F**), chemokine- (**G**) and inflammation-related pathways (**H**). All transcripts were ranked by the log2(fold change) between TKB-BKVN and TKB-STA. Each run was performed with 1,000 permutations. Differences in pathway activities scored by GSEA between TKB-BKVN and TKB-STA (**I**, **J**). Enrichment results with significant differences between TKB-BKVN and TKB-STA are shown. The functions of the pathways are shown in the annotations. (**K**) Heatmap of core genes in significantly enriched pathways between TKB1-BKVN and TKB1-STA. (**L**) Heatmap of core genes in significantly enriched pathways between TKB2-BKVN and TKB2-STA. In the heatmaps, blue means downregulation, while red means upregulation.

Based on the results of GSEA of the renal parenchyma, we found immune cell-related pathways (Figure 5A, 5E), such as positive regulation of CD4-positive alpha-beta T cell activation and CD4-positive or CD8-positive alpha-beta T cell lineage commitment; cytokine-related pathways (Figure 5B, 5F), including chemokine-related pathways (Figure 5C, 5G), such as chemokine activity and CXCR chemokine receptor binding; and inflammatory response-related pathways (Figure 5D, H), such as positive regulation of the acute inflammatory response and production of molecular mediators involved in the inflammatory response, were significantly enriched in TKB1-BKVN and TKB2-BKVN (all ES> 0; P <0.05). Figure 5I, 5J shows more comprehensive differences in enriched pathways. In addition to the above pathways, APP-related pathways were significantly upregulated in TKB1-BKVN and TKB2-BKVN (all ES> 0; P <0.05), whereas metabolism-related pathways, such as the metabolism of lipids, the lipid biosynthesis process, transferase activity, transferring acyl groups other than amino-acyl groups, and glucose homeostasis; and kidney development-related pathways were significantly downregulated in TKB1-BKVN and TKB2-BKVN (all ES <0; P <0.05). Heatmaps show the differences in the expression of core genes in the significantly enriched pathways shown in Figure 5A–5H and indicate that the T cell-, cytokine- (such as IFN-γ, IL-1, IL-6 and TNF), chemokine- and inflammation- (such as acute and chronic) related pathways were enriched in TKB-BKVN (Figure 5K, 5L).

### Differences in enriched pathways in the PB between BKV and STA

**Figure 6:**
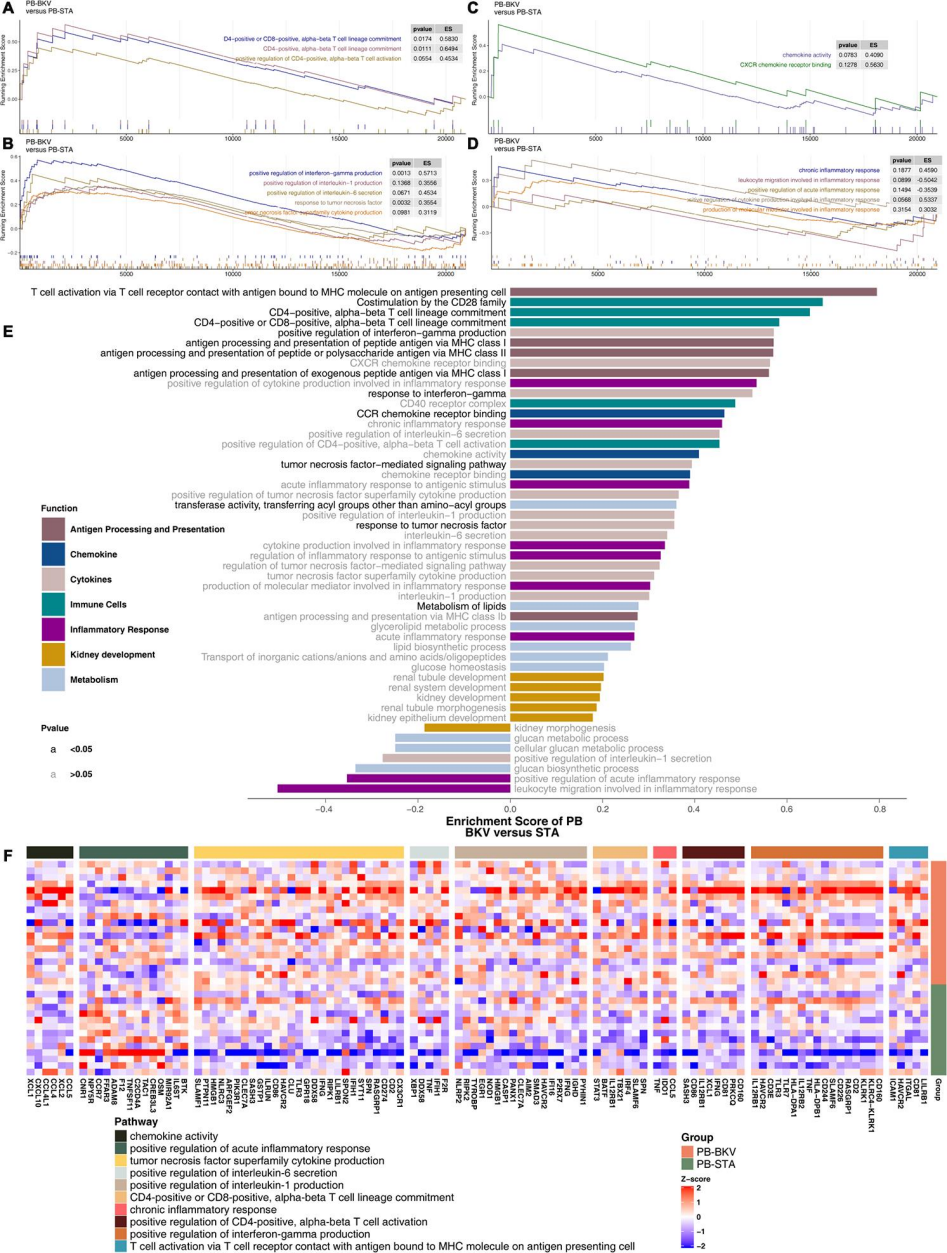
**GSEA of hallmark gene sets in PB-BKV and PB-STA data downloaded from MSigDB (GSE47199 and GSE75693).** GSEA results for immune cell- (**A**), cytokine- (**B**), chemokine- (**C**) and inflammation-related pathways (**D**). All transcripts were ranked by the log2(fold change) between PB-BKV and PB-STA. Each run was performed with 1,000 permutations. (**E**) Differences in pathway activities scored by GSEA between PB-BKV and PB-STA. Enrichment results with significant differences between PB-BKV and PB-STA are shown. The functions of the pathways are shown in the annotations. The black font indicates P < 0.05. The gray font indicates P > 0.05. (**F**) Heatmap of core genes in enriched pathways (the same as those in Figure 4K, 4L) between PB-BKV and PB-STA.

Based on the significantly different pathways identified by GSEA of the renal parenchyma, we analyzed whether PB-BKV and PB-STA also have the same trends in different pathways. The results indicated that pathways may differ in the renal parenchyma between PB-BKV and PB-STA. There were no significant differences in immune cell-related pathways, such as positive regulation of CD4-positive alpha-beta T cell activation (Figure 6A); cytokine-related pathways, such as positive regulation of IL-1 production, positive regulation of IL-6 secretion, and TNF superfamily cytokine production (Figure 6B); chemokine-related pathways (Figure 6C); or inflammatory response-related pathways (Figure 6D) between PB-BKV and PB-STA. Figure 6E shows a more comprehensive view of the differences in the GSEA-identified pathways, but there were no significant differences in most cytokine-, chemokine-, metabolism- and renal development-related pathways (ES <0; P> 0.05). In addition, the differences in the expression of core genes in the corresponding pathways (which significantly differed between TKB-BKVN and TKB-STA) in the PB also suggested that there were no significant differences in these pathways between PB-BKV and PB-STA (Figure 6F).

### Difference in ssGSEA scores in the renal parenchyma between BKVN and STAs and in the PB between BKV and STAs

**Figure 7:**
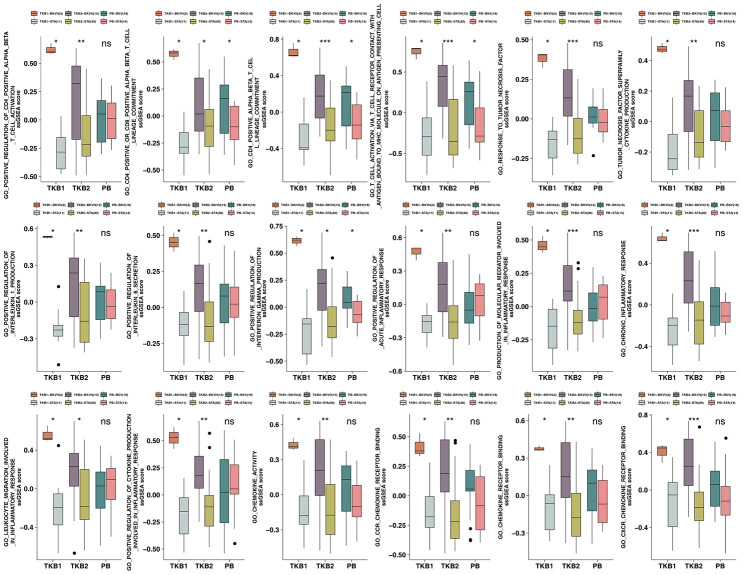
**ssGSEA scores for the PB (STA and BKV), TKB1 (STA and BKVN) and TKB2 (STA and BKVN) datasets.** The thick line represents the median value. The bottom and top of the boxes indicate the 25^th^ and 75^th^ percentiles (interquartile range). The whiskers encompass 1.5 times the interquartile range. *, P< 0.05; **, P< 0.01; ***, P< 0.001; and ****, P<0.0001; Mann-Whitney U test.

To further verify the differences in the activation levels of the above pathways in the renal parenchyma between BKVN and STAs and in the PB between BKV and STAs, ssGSEA was used to calculate the scores for the corresponding pathways for each sample. The results showed that the ssGSEA scores of TKB1-BKVN and TKB2-BKVN were similar. The ssGSEA scores for immune cell-related pathways (such as positive regulation of CD4-positive alpha-beta T cell activation and CD4- positive or CD8-positive alpha-beta T cell lineage commitment), TNF superfamily cytokine production, and positive regulation of IL-1 production were significantly higher for the BKVN renal parenchyma than for the STA renal parenchyma (all P <0.05; Figure 7), whereas the ssGSEA scores of most pathways, except for some immune cell- and IFN-γ-related pathways, were not significantly different between PB-BKV and PB-STA (Figure 7).

## DISCUSSION

**Figure 8:**
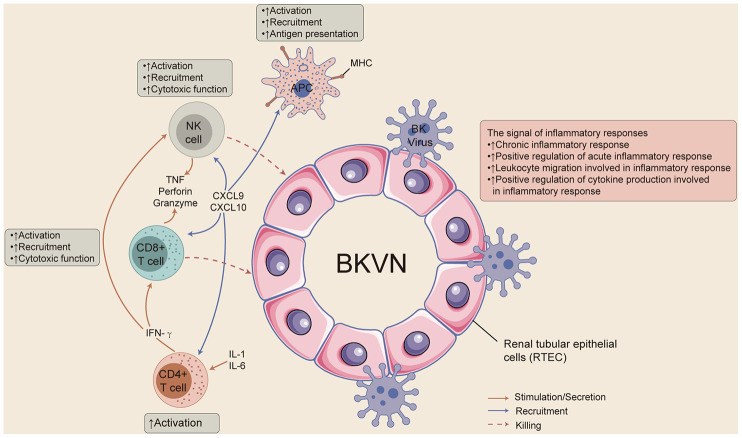
**The possible immune microenvironment in BKVN.**

In this study, we aimed to elucidate the unique immune microenvironment and special activated biological pathways in BKVN. We found that BKVN had an inflammatory immune microenvironment, as indicated by strong infiltration of activated memory CD4 T cells and significantly enriched immune cell- (CD4-positive alpha-beta T cell activation and CD4-positive or CD8-positive alpha-beta T cell lineage commitment), cytokine- (inflammatory factors) and chemokine-related pathways. Immunostaining showed that CD4+ and CD8+ cells were significantly high in BKVN compared to STA. Additionally, the renal parenchyma of BKVN had higher expression of APP-, CYT-, cytokine- and chemokine-related genes than the STA renal parenchyma. We also tried to compare the immune characteristics of BKVN and BKV in the PB. The proportion of plasma cells was higher in PB-BKV than in PB-STA, whereas the fraction of naive CD4 T cells was lower in PB-BKV than in PB-STA. Although there was a significant difference in the expression of CYT-related genes between PB-BKV and PB-STA, there were no significant differences in the expression of APP-, cytokine- or chemokine-related genes between PB-BKV and PB-STA. GSEA and ssGSEA results showed that the corresponding pathways significantly differed between TKB-BKVN and TKB-STA (including chemokine-, cytokine-, and inflammation-related pathways) but did not differ significantly between PB-BKV and PB-STA. Finally, we attempted to summarize the underlying immunological mechanism involved in the evolution of BKVN, which may lay the foundation for further prevention and treatment of BKVN (Figure 8).

Immune and nonimmune cells express viral receptors that allow them to recognize viral nucleic acids and/or viral proteins. Activation of these receptors can cause the release of proinflammatory mediators, chemokines, antiviral factors, and proapoptotic mediators to activate the natural immune response and specific immune responses to limit viral replication [1]. However, recent studies have shown that immune and inflammatory responses play roles in the occurrence and development of BKVN; for example, BK virus may cause BKVN through cell lysis, stimulating the immune system and inducing inflammation [1, 7, 10–12, 16]. Both GSEA results and ssGSEA results suggest that inflammation- and immune-related pathways are significantly enriched in BKVN. Therefore, it is particularly important to understand the underlying immune microenvironment in BKVN.

Studies have shown that certain immune cells, such as CD8+ T cells and NKs, may cause allograft loss in BKVN [10, 11]. For example, BKV-specific CD8+ T cell numbers are significantly higher in KTRs with a higher BK virus load than in those with a lower load, and all BKV-specific CD8+ T cells eventually contribute to the loss of allografts in KTRs [10]. The effective mediators of CD8+ T cells that perform cell killing are perforin (PFP), TNF, and Granzyme, which are also important markers for assessing CYT [17]. Furthermore, the expression of CD8A, GZMB, CD8B, GZMA and PRF1 can be used to assess CYT [18]. We found that the expression of CYT-related genes in TKB-BKVN was significantly higher than that in TKB-STA. Another study showed that the NKG2D receptors of NKs and cytotoxic T cells can play a role in BKVN by binding to PTX 3 (a cytokine-inducing protein related to innate immune responses and inflammation) and MICB (MHC-I class-related) [11]. Our results also showed that BKVN had strong infiltration of memory CD4 T cells and significantly activated immune cell-related pathways.

In addition to immune cells, cytokines and chemokines also play roles in BKVN [1, 16]. It has been reported that the levels of the proinflammatory cytokines IL-6, IL-8, TNF-α, and TGF-β are significantly increased in BKVN [19]. Our results also indicated that BKVN had significant activation of cytokine-related pathways (such as pathways involving IL-1, IL-6, TNF, IFN-γ, etc.). Additionally, the heatmap of cytokine-related pathways also showed that BKVN was associated with increased expression of cytokine-related genes. In addition, chemokines, as important components of the immune system, activate immune cells and recruit these cells to infected and/or inflamed tissues by binding to corresponding receptors [16]. Studies suggest that high expression of CCL5, CCL2, CXCL8, and CXCL10 may be related to the occurrence of BKVN. CXCL9 and CXCL10 can recruit T cells and NKs by combining with CXCR3 to further induce immune and inflammatory responses [20]. Our results showed that chemokine-related pathways were highly enriched in BKVN. Further exploration of core genes in these pathways revealed that the expression of CXCL9, CXCL10, CCL5, and CXCL8 in TKB-BKVN was significantly higher than that in TKB-STA.

Here, we present a comprehensive profile of the immune environment in BKVN based on bulk transcriptional analysis using microarrays. By comparing with stable allografts samples, we reveal an excessively immunosuppressive environment in BKVN. In the absence of BKVN specific antiviral therapy, active BK virus replication screening in the post-transplantation period is an essential prophylactic procedure to prevent graft damage [5]. It allows for the preemptive reduction of immunosuppressive therapy in the case of the detection of significant BKV and the prevention of the development of clinically significant nephropathy [21]. The goal of reducing immunosuppression is to prevent viral replication without inducing the development of rejection, although the optimal procedure for the stepwise reduction in immunosuppressive therapies remains unclear [21].

Although this study analyzed the immune microenvironment in the BKVN renal parenchyma and BKV PB and tried to clarify the potential immune-related mechanism involved in the development of BKVN. Our analyses, however, have several limitations: Firstly, our study only reveals relative changes in immune cells. While conventional ‘bulk’ methods (such as microarrays) cannot reflect the types and status at the single-cell level, only the average gene expression, which neglects the heterogeneity of the transcriptome at single-cell resolution, single-cell RNA-sequencing can reveal changes that render each individual cell type unique. Second, this study included only two kidney transplant cohorts so there may be bias in the evaluation of the immune microenvironment in the BKVN renal parenchyma and BKV PB. Third, rejection reactions mediated by T cells, B cells, inflammatory cytokines, and some chemokines are well-known confounders of BKVN, and we cannot exclude rejection as a confounding factor through this bioinformatic study. Finally, animal and laboratory experiments are mandatory to further clarify the role of the immune microenvironment in the pathogenesis of BKVN.

In summary, we investigated the role of the immune microenvironment in the pathogenesis of BKVN by evaluating immune cells, immune-related genes, and physiologically relevant pathways. The renal parenchyma of BKVN has an inflammatory immune microenvironment, including strong infiltration of activated memory CD4 T cells, increased expression of immune-related genes (such as cytokines, chemokines and CYT-related factors), and significant enrichment of immune- and inflammation-related pathways. Additionally, the differences in the immune microenvironment between PB-BKV and PB-STA may be different from those between TKB-BKVN and TKB-STA.

## MATERIALS AND METHODS

### Transplant kidney data

To explore the differences between BKVN and STAs and between BKV and STAs in terms of immune characteristics, we downloaded microarray data (GSE47199 and GSE75693) from the NCBI Gene Expression Omnibus (GEO) database. The expression data of GSE47199 and GSE75693 are annotated with the corresponding probes on GPL6244 and GPL570, respectively. The “normalizeBetweenArrays” function in the “limma” R package was used to normalize mRNA expression data [22]. For all samples in each dataset, probes for the same gene were reduced to a single value according to the maximum value [23]. The grouping information for the datasets (GSE47199 is divided into transplant kidney biopsy (TKB) 1 and peripheral blood (PB); GSE75693 is called TKB2) and the data processing procedure are detailed in Supplementary Figure 1.

### Analysis of immune cells and immune-related genes

The CIBERSORT algorithm can deconvolve the expression matrix of human leukocyte subtypes based on the principle of linear support vector regression [13]. Based on known gene expression feature sets (including 547 gene tags; the LM22 gene set), we inferred the proportions of cell types in an expression matrix of mixed cell types. We used the CIBERSORT web portal (http://cibersort.stanford.edu/) to analyze the abundances of 22 immune cell types in GSE47199, GSE75693. The cell markers for different immune cells were taken from Newman et al. [13]. In addition, antigen processing and presentation- (APP), cytotoxicity- (CYT), chemokine- and cytokine-related genes were taken from Thorsson et al. and Rooney et al. [14, 15].

### Immunohistological analysis

Renal allograft biopsy was obtained from patients with BKVN and STA at Zhujiang Hospital of Southern Medical University, Guangzhou, China. Informed consent was obtained from all patients, and the Human Subjects Committee of Zhujiang Hospital of Southern Medical University approved all of the study protocols. Among them, five recipients were assigned to the BKVN, while ten recipients were assigned to the STA. The baseline patient characteristics and level of CD4+ and CD8+ cells are listed in Supplementary Table 1. Formalin-fixed, paraffin-embedded renal biopsy sections (n=15) were deparaffinized in xylene and rehydrated in graded ethanol (100%–95%), treated by 3% hydrogen peroxide for 10 min to inhibit the endogenous peroxidase, added with anti-CD4 and -CD8, respectively. The status of CD4+ and CD8+ cells in BKVN and STA were recorded: - (no cells staining), +/- (1-5% cells staining), 1+ (5-10% cells staining), 2+ (10-50% cells staining), or 3+ (>50% cells staining). Additionally, the findings were divided: CD4 positive (+;++;+++) and CD4 negative (-;-/+); CD8 positive (+;++) and CD8 negative (-;-/+).

### Gene enrichment analysis (GSEA) and single-sample GSEA (ssGSEA)

The gene expression data in GSE47199 and GSE75693 were normalized by the “limma” R package for GSEA. Using the clusterProfiler R package and The Molecular Signatures Database (MSigDB) to annotate the gene expression data, P< 0.05 in gene ontology (GO) (biological process, BP), GO (molecular function, MF), GO (cellular component, CC), Kyoto Encyclopedia of Genes and Genomes (KEGG) and Reactome analyses was considered to be indicate significantly different pathways. Enrichment scores and P values were based on 1,000 permutations. In addition, the data were annotated using the GSVA R package and MSigDB gene sets (c2.all.v7.0.symbols.gmt and c5.all.v7.0.symbols.gmt) to perform ssGSEA on the expression data of each sample in GSE47199 and GSE75693 [24].

### Statistical analysis

The Mann-Whitney U test was used to compare the proportion of immune cells, the expression of cell marker genes, the expression of immune-related genes, and ssGSEA scores between different groups (TKB1: BKVN versus STAs; PB: BKV versus STAs; and TKB2: BKVN versus STAs). Fisher's exact test was used to compare the status of CD4+ and CD8+ between BKVN and STA from Zhujiang Hospital of Southern Medical University. P <0.05 was considered statistically significant. All statistical tests were two sided. All statistical tests and visualization analysis were completed with R software (version 3.6.1). The “gseaplot2” function in the R package clusterProfiler was used to visualize the pathways identified by GSEA. The R package Complexheatmap was used to create heatmaps [25].

## Supplementary Material

Supplementary Figures

Supplementary Table 1
