# Supplementary material for: Characterization of aberrant pathways activation and immune microenviroment of BK virus associated nephropathy
Source: Aging (Albany NY). 2020 Jul 13;12(14):14434–51. doi: 10.18632/aging.103486 (PMC7425495; doi:10.18632/aging.103486)
Supplement: Supplementary Figures [file aging-12-103486-s001..pdf]

SUPPLEMENTARY TABLE

Supplementary Table 1. The clinical and immunohistochemical features (CD4 and CD8) of patients from Zhujiang Hospital of Southern Medical University (n=15).

Patient ID	Disease	cd8	cd4	Gender	Age (years)	CD8	CD4
Patient8	STA	+/-	+/-	Female	21	Negative (-;-/+)	Negative (-;-/+)
Patient9	STA	+	+	Female	22	Positive (+;++)	Positive (+;++;+++)
Patient10	STA	+/-	+/-	Male	40	Negative (-;-/+)	Negative (-;-/+)
Patient7	STA	-	-	Female	23	Negative (-;-/+)	Negative (-;-/+)
Patient11	STA	+/-	+/-	Male	31	Negative (-;-/+)	Negative (-;-/+)
Patient12	STA	+	++	Female	42	Positive (+;++)	Positive (+;++;+++)
Patient13	STA	+	++	Female	53	Positive (+;++)	Positive (+;++;+++)
Patient14	STA	+/-	+/-	Female	33	Negative (-;-/+)	Negative (-;-/+)
Patient15	STA	+/-	+	Female	29	Negative (-;-/+)	Positive (+;++;+++)
Patient6	STA	-	-	Male	35	Negative (-;-/+)	Negative (-;-/+)
Patient1	BKVN	++	++	Female	40	Positive (+;++)	Positive (+;++;+++)
Patient3	BKVN	++	+	Male	33	Positive (+;++)	Positive (+;++;+++)
Patient4	BKVN	+	+	Male	48	Positive (+;++)	Positive (+;++;+++)
Patient5	BKVN	+	++	Female	32	Positive (+;++)	Positive (+;++;+++)
Patient2	BKVN	++	+++	Male	34	Positive (+;++)	Positive (+;++;+++)
