# Supplementary material for: Characterization of aberrant pathways activation and immune microenviroment of BK virus associated nephropathy
Source: Aging (Albany NY). 2020 Jul 13;12(14):14434–51. doi: 10.18632/aging.103486 (PMC7425495; doi:10.18632/aging.103486)
Supplement: Supplementary Table 1 [file aging-12-103486-s002..pdf]

SUPPLEMENTARY FIGURES

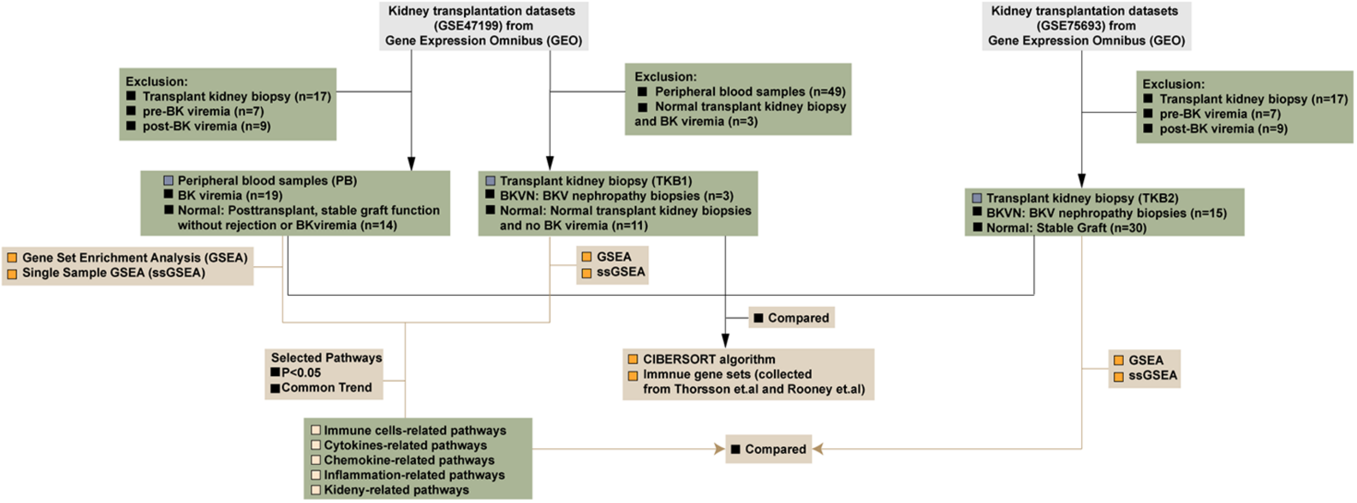

Supplementary Figure 1. Flow chart of this study.

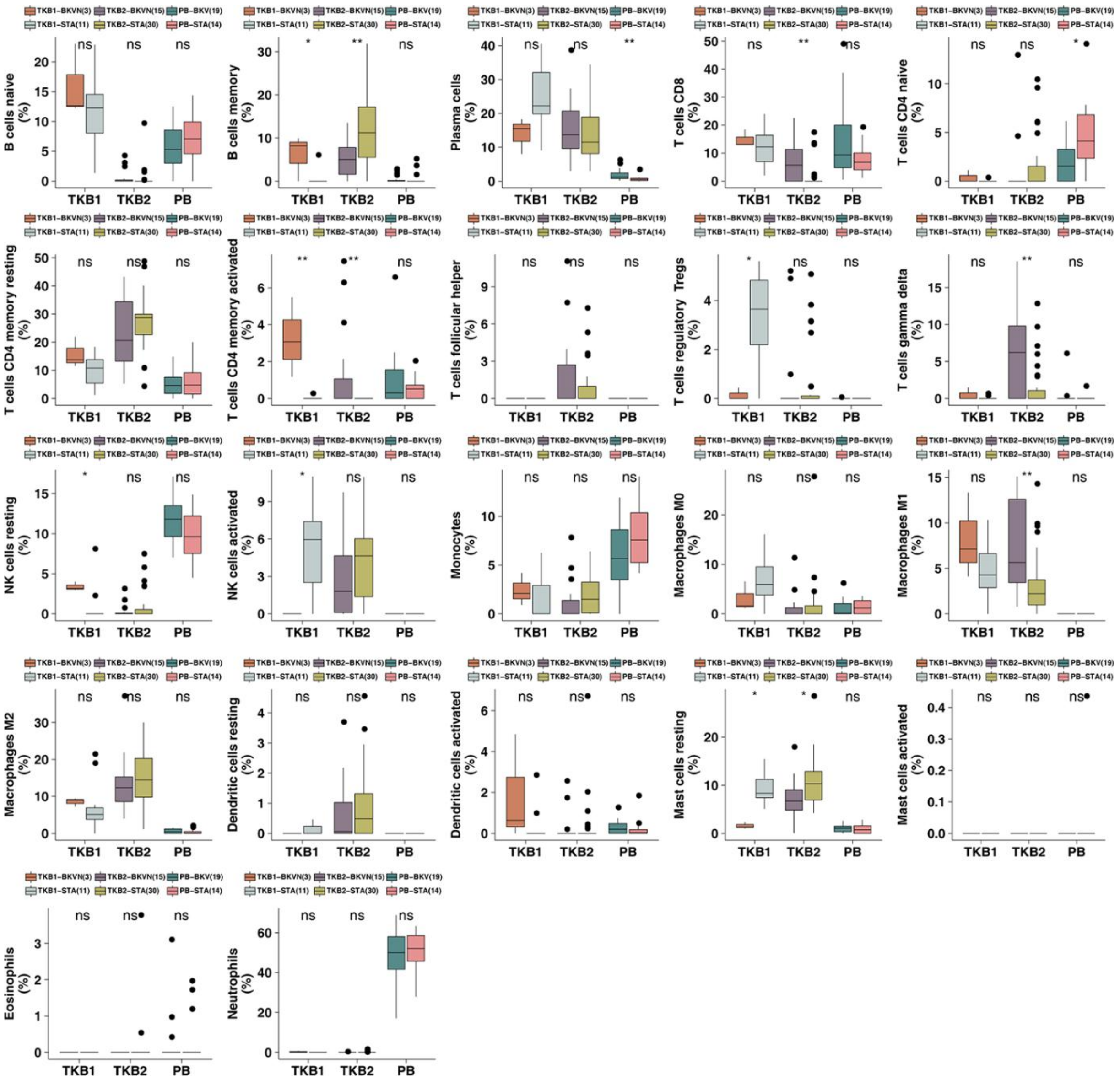

Supplementary Figure 2. The proportions of immune cells in the PB (STA and BKV), TKB1 (STA and BKVN) and TKB2 (STA and BKVN) datasets.

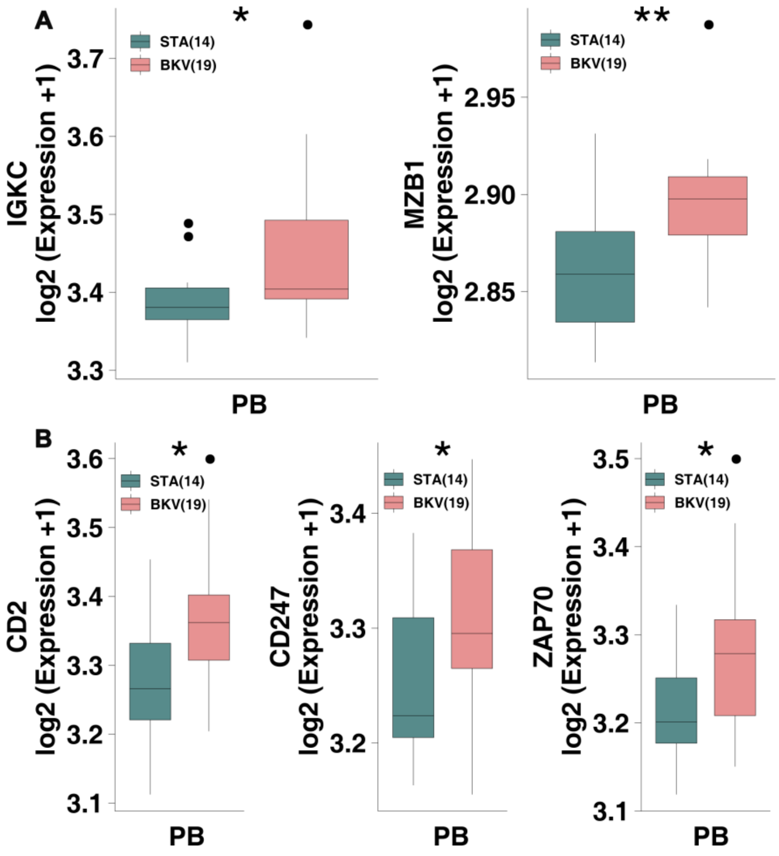

Supplementary Figure 3. Comparisons of the cell markers of plasma cells (**A**) and naive CD4 T cells (**B**) between PB-STA and PB-BKV.
